# Intragastric rupture of a splenic artery aneurysm - a case report

**DOI:** 10.1186/1757-1626-2-202

**Published:** 2009-11-18

**Authors:** Abdelmalek Ousadden, Karim H Ibnmajdoub, Hicham Elbouhaddouti, Khalid Mazaz, Khalid AitTaleb

**Affiliations:** 1 Service de chirurgie générale - Hôpital des spécialités - CHU de Fès - Route de Sidi Harazem - Fès - 30070 - Morocco

## Abstract

**Introduction:**

Hematemesis caused by intragastric rupture of a splenic artery aneurysm, is an exceptional and potentially lethal emergency.

**Case presentation:**

A 36 years old woman, mother of seven children presented with hematemesis. The gastric endoscopy revealed a bleeding polypoid lesion leading to a surgical management. The operative discovery of a complicated splenic artery aneurysm, led to its resection with splenectomy and gastric suture.

**Conclusion:**

Intragastric rupture of a splenic artery aneurysm is an exceptional emergency which urgent diagnosis and management can avoid a potential lethal evolution.

## Introduction

Splenic artery aneurysm (SAA) is the most frequent visceral artery aneurysm (60% of all cases) and the third most common site of intra-abdominal aneurysm after aorta and iliac arteries [1,2]. Its incidence is 0.01-0.2% [3]. A gastric bleeding and hematemesis exceptionally reveal SAA.

## Case presentation

**Figure 1:**
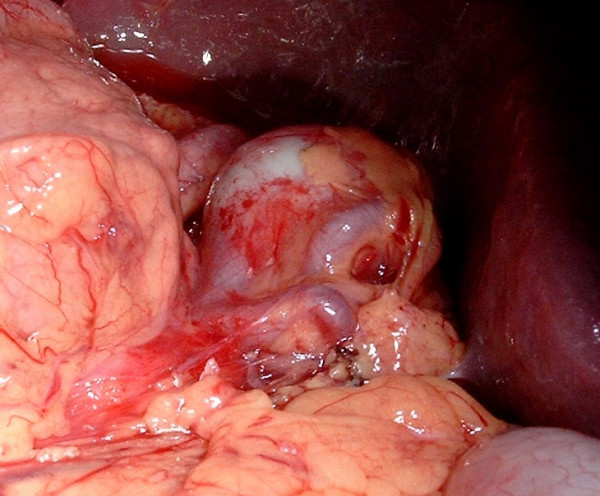
**Splenic artery aneurysm located between the spleen and stomach**.

**Figure 2:**
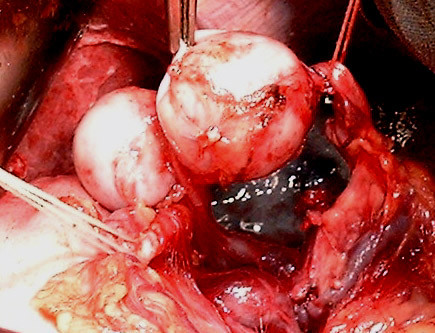
**Splenic artery aneurysm released from the posterior surface of the stomach, the splenic vein and hilum of the spleen**.

**Figure 3:**
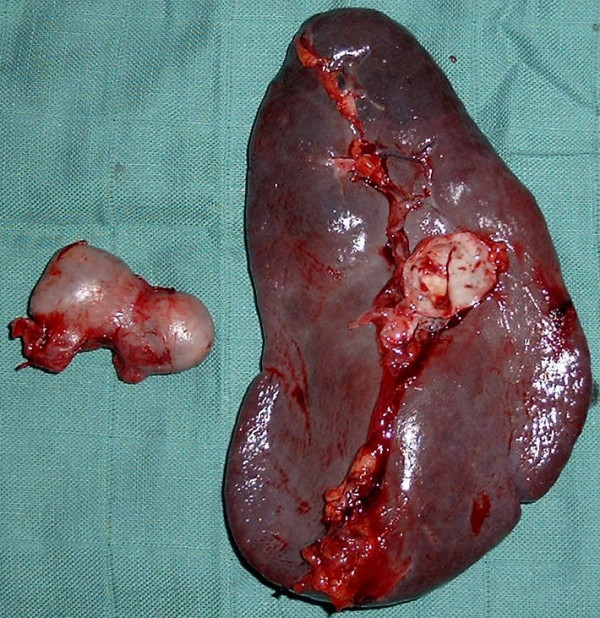
**Resected spleen and aneurysm**.

A 36 years old woman, mother of seven children, was admitted in the emergencies for hematemesis. Two episodes 10 days and 2 days before admission were reported, accompanied with epigastric pain. The gastric endoscopy found a polypoid-bleeding lesion. Biological tests and abdominal ultrasonography were normal. A laparotomy for haemostasis was made. An examination of the gastric mucosa after gastrotomy did not find the lesion described in fibroscopy. However a splenic artery aneurysm was individualized. It was adherent to the posterior side of the stomach from which it was released. The diagnosis of SAA fistulation through the posterior wall of the stomach was made. This 2-4 cm diameter aneurysm was sacciforme, multiple and joining the spleen hilum (Figure 1) without other abdominal aneurysm location. A gastric suture, an aneurysm resection and a splenectomy were then performed (Figures 2 and 3). A pneumococcal vaccine and an antibiotic prophylaxis have been made and there were no postoperative complications. Post-operative fibroscopy and visceral arteries imaging were normal.

## Discussion

The SAA is more frequent in women (nearly 80% of all cases) [4], especially during pregnancy or in multiparous [2,4,5]. During pregnancy, high splenic blood flow and increased hormone levels (oestrogen, progesterone, relaxine), cause a deleterious effect on elastic tissue of the splenic artery [3,5]. These effects are cumulative with each successive pregnancy [5]. Our patient had seven pregnancies. Only 20% of all SAA are multiple [2,6] as in our case. They are most often saccular, exceptionally fusiform and are located in about 80% on the distal third of the artery [2].

The SAA diagnosis is difficult cause only 17% of all patients are symptomatic [6,7]. These symptoms are non-specific [4]. Abdominal radiograph shows rarely a prevertebral calcification. For diagnosis, ultrasonography, pulsed Doppler, computed tomography and magnetic resonance imaging are useful, when arteriography is the gold standard [4]. The principal complication of a SAA >2 cm is rupture [1,4,6,8]. The reported risk varies from 2 to 9.6% [6,8]. The rupture happens sometimes in the in the splenic vein and often in the peritoneal cavity causing a cataclysmic bleeding. In 30% of all cases, the rupture occurs in a viscera [8] like the pancreas, the colon or the stomach [4] causing a digestive bleeding as in our case. To date, within more than 3000 reported digestive artery aneurysms, there are fewer than 100 SAA intragastric ruptures [9].

The SAA rupture causes 25% average mortality [5,6], about 70% during pregnancy and 95% foetal mortality [1]. These serious risks justify a SAA management. This management can be made by Interventional radiological techniques (arterial stent or percutaneous angiographic embolization) [3,10] or by surgery (operative occlusion, resection or arterial bypass) [3,9]. The treatment must be the most conservative for the spleen. But distal localisation, near the spleen hilum, as in our case, can impose a splenectomy [3]. A gastro-aneurismal fistula can be managed by gastric suture.

## Conclusion

The intragastric rupture of a spelnic artery aneurysm is exceptional. Its presentation as a digestive bleeding is an emergency which urgent management can avoid a potential lethal evolution.

## Consent

Written informed consent was obtained from the patient for publication of this case report and accompanying images.

## Competing interests

The authors declare that they have no competing interests.

## Authors' contributions

AO, KA and KM operated on the patient. KHI took the photos. HE participated in following up. All authors participated in writing the case report. All authors read and approved the final manuscript.
